# Integrative Pan-Cancer Analysis Confirmed that FCGR3A is a Candidate Biomarker Associated With Tumor Immunity

**DOI:** 10.3389/fphar.2022.900699

**Published:** 2022-05-20

**Authors:** Lilin Li, Zijian Huang, Kunpeng Du, Xiang Liu, Chunhui Li, Duanyu Wang, Yangfeng Zhang, Changqian Wang, Jiqiang Li

**Affiliations:** ^1^ Department of Radiation Oncology, Oncology Center, Zhujiang Hospital of Southern Medical University, Guangzhou, China; ^2^ Department of Oncology, The First Affiliated Hospital of Guangdong Pharmaceutical University, Guangzhou, China

**Keywords:** pan-cancer analysis, FCGR3A, tumor biomarker, immune infiltration, tumor microenvironment, prognosis, drug sensitivity

## Abstract

**Background:** Fc gamma receptor 3A (FCGR3A) encodes a receptor for the Fc portion of immunoglobulin G, which plays a significant role in the immune response. However, the role of FCGR3A in cancers remains unclear. This study aimed to visualize the prognostic landscape of FCGR3A in pan-cancer and investigate the relationship between FCGR3A expression and tumor microenvironment.

**Method:** Based on the TCGA database, GTEx database, and GDSC database, we analyzed the expression of FCGR3A in pan-cancers and adjacent normal tissues and its relationship with prognosis, immune cells infiltration, immune-related genes, DNA mismatch repair (MMR) genes, DNA methylation, and drugs sensitivity. The gene alteration frequency of FCGR3A was acquired on the cBioportal website. Moreover, we constructed PPI networks, performed GO and KEGG analysis to illustrate the function, and signaling pathways of FCGR3A-related genes, and conducted gene set enrichment analysis (GSEA) of FCGR3A to further explore its potential biological functions.

**Result:** The differential analysis results of the publicly available databases showed that FCGR3A was generally highly expressed in pan-cancer. Survival analysis revealed that FCGR3A predominated as a risk prognostic factor in most cancers. Additionally, the expression of FCGR3A was confirmed to be associated with several immune cells infiltration, multiple immune checkpoint genes, and DNA mismatch repair genes expression in generalized carcinoma. We also identified a negative correlation between FCGR3A and DNA methylation levels. Through GO/KEGG and GESA, we found that FCGR3A was involved in many pathologic and physiological processes, and was most closely related to tumor immune-related pathways. Drug sensitivity analysis showed that higher FCGR3A expression predicts a low IC50 value for the vast majority of drugs.

**Conclusions:** FCGR3A may be an immune-oncogenic molecule that correlates with tumor immune infiltration levels and affects drug sensitivity, thus it can be served as a promising biomarker for cancer detection, prognosis, therapeutic design, and follow-up.

## Introduction

Malignant tumor is one of the main causes of death in the world and a major obstacle affecting the quality of human life. So far, there is no absolute cure for cancer (Bray et al., 2018). Tumor biomarkers can be used for early detection, diagnosis, therapeutic targets, response prediction, treatment monitoring, prognosis determination, and personalized combination therapy. More and more tumor biomarkers, such as PD-L1, BRCA1/2, BRAF, HER2, etc., have been discovered, which become an indispensable tool in current tumor treatment due to its ability to assist various clinical decisions (Zhou et al., 2015). However, most targeted or immunotherapy has limited efficacy, so it is necessary to discover more tumor markers and study their role and value in generalized cancer, evaluate their correlation with clinical prognosis and related signaling pathways, in order to accurately predict prognosis and provide options for tumor treatment.

There are two FcγRIII genes in the human genome, one encodes FcγRIIIa and the other encodes FcγRIIIb. These two proteins share 97% homology at the amino acid level (Gessner et al., 1995). FCGR3A gene encodes the FcγRIIIa receptor in most effector cells such as macrophages, NK, and γδ T cells, and possesses a low affinity for IgG-containing immune complexes (IC). Human FcγRIIIa (CD16a), a type I transmembrane protein, is an extensively glycosylated heterogeneous protein, the Fc-fragment is recognized through loops of the C-terminal receptor domain of the FcγRIII, transmitting activating signals in effector cells (Sondermann et al., 2001). Mutations in the FCGR3A gene are linked to recurrent viral infections, susceptibility to systemic lupus erythematosus, and alloimmune neonatal neutropenia (Cartron et al., 1991; Patel et al., 2020).

FCGR3A is involved in the removal of antigen-antibody complexes from the circulation, as well as other antibody-dependent responses (Vance et al., 1993; Hargreaves et al., 2015). Activation of FcγRIIIa on NK cells plays a key role in mediating antibody-dependent cell-mediated cytotoxicity (ADCC), while activation of FcγRIIIa on macrophages is important in mediating antibody-dependent cellular phagocytosis (ADCP) (Morvan and Lanier, 2016), which are the innate immune mechanism that eliminates cancer cells. They can be used to treat a variety of cancers that overexpress unique antigens, such as neuroblastoma, breast cancer, B cell lymphoma, etc. (Pandey and Namboodiri, 2014; Ziakas et al., 2016; Musolino et al., 2022)

In addition, there is a G559T polymorphism in the FCGR3A gene, whose two common alleles encode two variants that differ at position 158, one Val (V158) or one Phe (F158). The binding affinity of FcγRIIIa to IgG varies with allele variants. Specifically, FcγRIIIa-V158 has a higher affinity to human IgG1 and IgG3 than does FcγRIIIa-158F, this stronger binding affinity results in more potent *in vitro* ADCC and tumor cell death (Koene et al., 1997). ADCC is a key effector mechanism of NK cells mediated by therapeutic monoclonal antibody (mAb). Better clinical outcomes have been observed in patients expressing high-affinity FcγRIIIa variant (V158) when they were treated with anti-CD20 or anti-EGFR antibodies (Veeramani et al., 2011). An over-representation of the FcγRIIIa-158F allele has been reported as a major risk factor for patients with systemic lupus erythematosus (SLE) (Edberg et al., 2002). In addition, FcγRIIIa polymorphisms influence clinical outcomes in colorectal cancer, squamous cell head and neck cancer, and ERBB2/HER2-positive breast cancer patients treated with anti-epidermal growth factor receptor (EGFR) antibodies such as rituximab, cetuximab, and trastuzumab: Patients with FCGR3A-157V/V genotypes had significantly longer survival (Calemma et al., 2012; Gavin et al., 2017; Magnes et al., 2018).

Although the FCGR3A-158 V-F polymorphism impacts multiple autoimmune and infectious diseases and affects the response of monoclonal therapy (mAb) in some tumor patients, there is insufficient scientific evidence regarding the pathogenic role of FCGR3A in diverse cancers and whether FCGR3A functions in the immune microenvironment of different tumors through certain common molecular mechanisms.

It is well known that cancer is a genetic disease, and even when patients are affected by apparently the same type of cancer, the mutant signature of the cancer type can vary from patient to patient. These genetic changes may affect the efficacy of anticancer drugs and affect the clinical response in tumor patients. For example, Venetoclax is effective in small cell lung cancer with high bcl-2 expression (Lochmann et al., 2018). However, for the vast majority of tumor types and available therapeutic agents, the genotype-phenotypic association between gene expression differences and anticancer drug responses is not simple (Vuong et al., 2014). Therefore, in this study, we used multiple databases to analyze FCGR3A gene expression, prognosis, immune infiltration correlation, and epigenetic status in pan-cancer, and to explore the underlying molecular mechanisms and its relationship with drug sensitivity, so as to evaluate the impact of FCGR3A on the tumor microenvironment.

## Materials and methods

### Expression Analysis of FCGR3A in Pan-Cancer

The expression difference of the FCGR3A gene in pan-cancer and their adjacent normal tissues were analyzed using the Sangerbox website (http://sangerbox.com/). Sangerbox is an open network containing tumor and normal samples data from The Cancer Genome Atlas (TCGA) (https://portal.gdc.cancer.gov/) and the Genotype-Tissue Expression (GTEx) database (https://gtexportal.org/). To further evaluate FCGR3A expression in pan-cancer, we also using Tumor Immune Estimate Resources (TIMER2.0) (http://timer.cistrome.org/) and Gene Expression Profiling Interactive Analysis (GEPIA2) (http://gepia2.cancer-pku.cn/#analysis) web server to obtain the FCGR3A expression prospect in TCGA datasets. In order to identify the tumor cell types in which FCGR3A is predominantly expressed, we downloaded the FCGR3A gene expression data in 886 tumor cell lines from the Genomics of Drug Sensitivity in Cancer (GDSC) database (https://www.cancerrxgene.org/). The R packages “ggplot2” and “ggpubr” were used to analyze and compare the expression of FCGR3A in different tumor cell lines. FCGR3A gene expression levels in pan-cancer single-cell samples were also obtained through the cancerSCEM website (https://ngdc.cncb.ac.cn/cancerscem/). In addition, the expression of FCGR3A at the protein level in different tumors was analyzed in the Clinical Proteomic Tumor Analysis Consortium (CPTAC) (https://pdc.cancer.gov/pdc/browse) and the Human Protein Atlas (HPA) database (http://www.proteinatlas.org/). The criterion for classifying tumor samples into high and low expression was the median FCGR3A expression. Kruskal-Wallis rank-sum test was applied to statistical analysis, with *p* < 0.05 being deemed statistically significant.

### Survival Prognosis Analysis

Expression data of various genes in pan-cancer were obtained from TCGA database and the GTEx database by UCSC Xena (http://xenabrowser.net/datapages/). Extraction of FCGR3A single-gene expression data with Strawberry Perl (http://strawberryperl.com/). Survival data also downloaded from UCSC Xena. To determine the relationship between expression of FCGR3A and survival prognosis, R package “survival” was performed to determine the correlation between FCGR3A mRNA expression with overall survival (OS), disease specific survival (DSS) and progression-free survival (PFS), and univariate Cox regression analysis was used as statistical method. It was described as forest plots using the R package “forestplot”. Furthermore, we used the “Stage Plot " module of the GEPIA2 website to obtain violin plots of FCGR3A expression in all TCGA tumors at different pathological stages.

### Immunological Correlation Analysis

Using the TIMER2.0 web server (http://timer.cistrome.org/) to acquire the correlation data of FCGR3A expression with infiltrating immune cells and the abundance of immune cell markers from the TCGA pan-cancer. The expression status of immune-related genes (including immunosuppressive genes and chemokines) in 33 cancers was obtained from the UCSC Xena website (http://xenabrowser.net/datapages/). R package “limma” was utilized for the purpose of investigating the relationship between FCGR3A expression and the expression level of immune-related genes, and the correlation coefficient was determined by Spearman’s correlation analysis. Visualization was carried out through the R “reshape2” and “RColorBreyer” packages.

### Genetic Alteration and DNA Mis-Match Repair Genes Correlation Analysis

In the cBioPortal website (https://www.cbioportal.org/), select the “TCGA Pan Cancer Atlas Studies” in the “Quick select” module, FCGR3A was input to query the characteristics of genetic change and obtain the change frequency, mutation type, and CNA (copy number alteration) results of all TCGA tumors. In addition, R package “limma” was used to estimate the correlation between the FCGR3A gene and the expression of four MMRs (MLH1, MSH2, MSH6, and PMS2), and the results were visualized as correlation heat map by R packages “reshape2” and “RColorBreyer”. The MMRs gene expression profiles of various tumors were derived from the TCGA database.

### DNA Methylation Correlation Analysis

Based on the TCGA database, methylation levels of different tumors and their corresponding normal tissues were analyzed using the “methylation” module of the UALCAN website (http://ualcan.path.uab.edu/), and boxplots of difference analysis were downloaded. Tumor samples were divided into high- and low-expression groups based on median FCGR3A expression. On the vertical axis, beta value ranging from 0 (unmethylated) to 1 (fully methylated) represent DNA methylation levels. A beta value of 0.7–0.5 is generally considered to be hypermethylation, while a beta value of 0.3–0.25 is hypomethylation (Men et al., 2017).

### FCGR3A-Related Gene Analysis

We used the STRING website (https://cn.string-db.org/) to obtain the available experimentally determined FCGR3A-binding proteins according to the following criteria: network type (“full STRING networks”), meaning of network edges (“evidence”), active interaction sources (“experiments, text mining, databases”), minimum required interaction score [“medium confidence (0.400)”], max number of interactors to show (“no more than 30 interactors” in 1st shell) and active interaction sources (“experiments”). Protein-protein interaction (PPI) networks were visualized using Cytoscape (version 3.8.2). Next, we conducted Gene Ontology (GO) and Kyoto Encyclopedia of Genes and Genomes (KEGG) enrichment analysis for the above FCGR3A-related genes using the R package “Cluster Profiler”, and visualized using the “ggplot2” package.

### Gene Set Enrichment Analysis of FCGR3A in Pan-Cancer

With the help of the R software package “clusterProfiler”, we performed GSEA based on the GO dataset to explore the biological function of FCGR3A in tumor progression. We selected eight types of cancer whose prognosis is associated with FCGR3A expression, and use the “enrichplot” package to show the top five signaling pathways most significantly enrichment enriched in the database.

### Drug Sensitivity Analysis

The data of gene expression level in pan-cancer cell lines and the drug sensitivity (IC50) of 265 compounds in these cell lines were downloaded from the Genomics of Drug Sensitivity in Cancer (GDSC) database (https://www.cancerrxgene.org/). Spearman’s-correlation analysis was used to explore the correlation between drug sensitivity and FCGR3A gene expression in 962 cancer cell lines. Then, all cell lines were divided into low expression and high expression groups according to the median FCGR3A expression level. Kruskal-Wallis rank-sum test was used to analyze the drug sensitivity (IC50) difference of six commonly used anticancer drugs, *p* < 0.05 was considered statistically significant.

## Results

### Expression Levels of FCGR3A in Pan-Cancer

**FIGURE 1:**
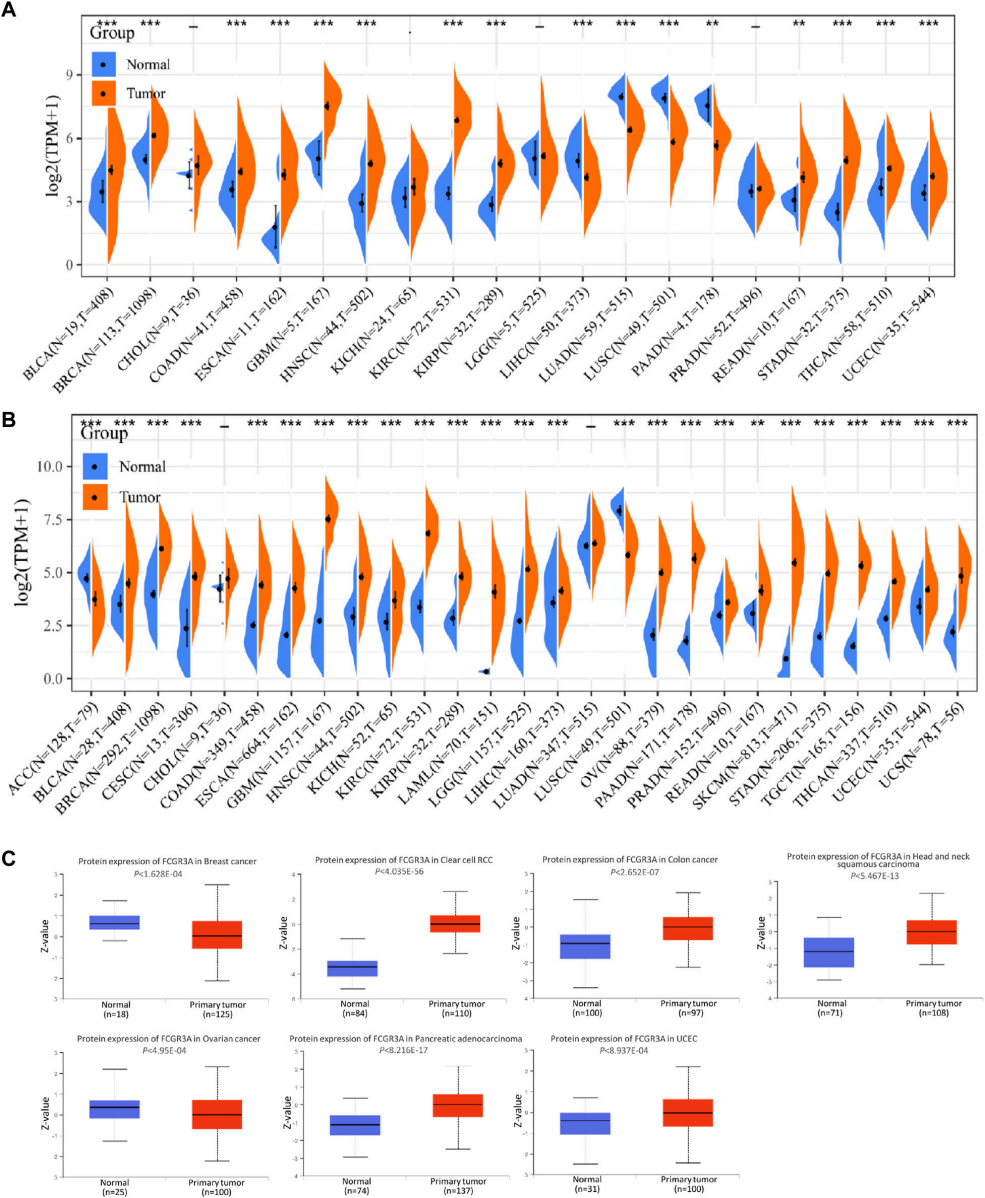
Expression level of FCGR3A gene in pan-cancer. **(A)** FCGR3A expression levels in tumors containing 20 TCGA tissues and paired adjacent non-cancerous tissues; **(B)** FCGR3A expression difference in 27 tumors integrating data of normal tissues in GTEx database and data of tumor tissues in TCGA database; **(C)** Based on the CPTAC dataset, the expression level of FCGR3A protein in normal and primary tumor tissues was analyzed. *p < 0.05; **p < 0.01; ***p < 0.001.

FCGR3A mRNA expression levels were analyzed by using different databases to detect FCGR3A expression across a wide range of cancers. The differential expression profile of FCGR3A in tumor and adjacent tumor tissues was retrieved from the TCGA database, as shown in Figure 1A. Considering the small number of normal samples in TCGA, we integrated data from the TCGA and GTEx database to conducted FCGR3A expression differential analysis in 27 tumors (Figure 1B), compared with the corresponding normal group, FCGR3A was generally overexpressed in the cancer group, including bladder urothelial carcinoma (BLCA), breast invasive carcinoma (BRCA), cervical squamous cell carcinoma and endocervical adenocarcinoma (CESC), colon adenocarcinoma (COAD), esophageal carcinoma (ESCA), glioblastoma multiforme (GBM), head and neck squamous cell carcinoma (HNSC), kidney chromophobe (KICH), kidney renal clear cell carcinoma (KIRC), kidney renal papillary cell carcinoma (KIRP), acute myeloid leukemia (LAML), brain lower grade glioma (LGG), liver hepatocellular carcinoma (LIHC), ovarian serous cystadenocarcinoma (OV), pancreatic adenocarcinoma (PAAD), Prostate adenocarcinoma (PRAD), rectum adenocarcinoma (READ), skin cutaneous melanoma (SKCM), stomach adenocarcinoma (STAD), testicular germ cell tumors (TGCT), thyroid carcinoma (THCA), and uterine corpus endometrial carcinoma (UCEC) and uterine carcinosarcoma (UCS). Meanwhile, a lower expression of FCGR3A was found in adrenocortical carcinoma (ACC) and lung squamous cell carcinoma (LUSC) dataset. To further verify the above results, we applied the TIMER and GEPIA2 website to obtain the expression status of FCGR3A across various cancer types of TCGA, and both showed that FCGR3A was highly expressed in most tumor tissues (Supplementary Figure S1). Furthermore, to identify tumor cell types that predominantly express FCGR3A, we analyzed FCGR3A gene expression levels in pan-cancer single cell lines using the GDSC database (Supplementary Figure S1A) and the CancerSCEM website (Supplementary Figure S1B), and the results showed that FCGR3A was highly expressed in most tumor cell lines. The top 5 FCG3A-expressing tumor cell lines in the GDSC database are chronic lymphocytic leukemia (CLL), TGCT, lymphoid neoplasm diffuse large B-cell lymphoma (DLBC), LAML, and mesothelioma (MESO), while the top 5 FCG3A-expressing tumor cell lines in the CancerSCEM database are GBM, lung cancer, PRAD, LAML, and coloretal cancer (CRC). Lastly, results from the CPTAC dataset showed that FCGR3A total protein expression was higher in primary tissues of breast cancer, clear cell renal cell carcinoma, colon cancer, head and neck squamous cell carcinoma, ovarian cancer, pancreatic adenocarcinoma, and uterine corpus endometrial carcinoma than in normal tissues. (Figure 1C).

**FIGURE 2:**
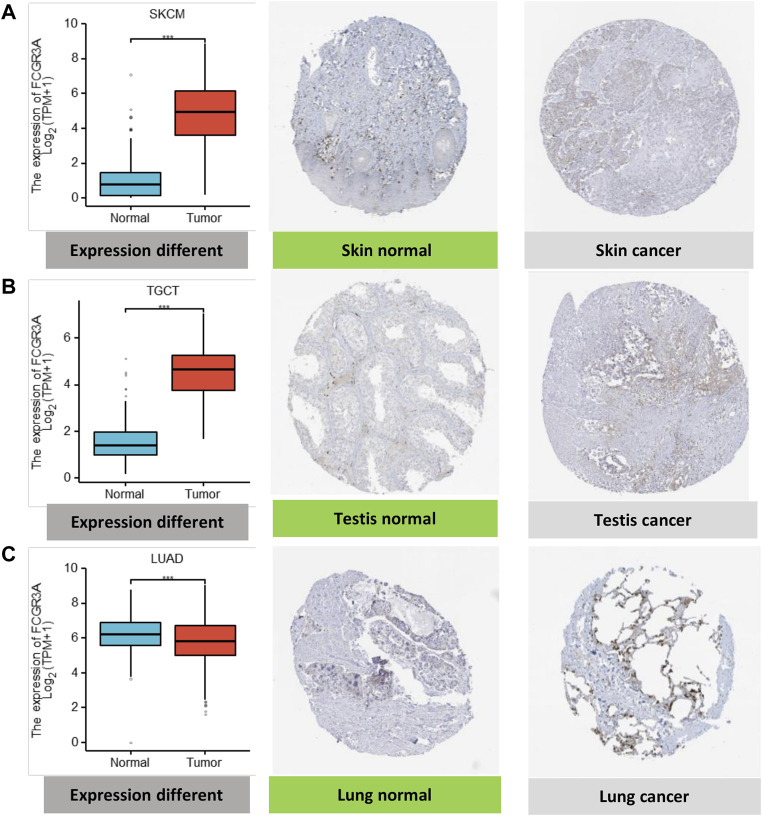
Comparison of FCGR3A gene expression between normal and tumor tissues (left) and immunohistochemistry images in normal (middle) and tumor (right) tissues. **(A)** Skin; **(B)** Testis; **(C)** Lung. *p < 0.05; **p < 0.01; ***p < 0.001.

Moreover, to assess FCGR3A expression at the protein level, we acquired immunohistonchemistry (IHC) results from the HPA database and compared the results with FCGR3A gene expression data from TCGA. As shown in Figures 2A–C, the data analysis results of the two databases were consistent. Normal skin and testis tissues showed not detected staining with FCGR3A IHC, while tumor tissues showed medium staining. In contrast, normal lung tissue showed strong FCGR3A staining and lung cancer showed weak FCGR3A staining.

### Prognostic Value of FCGR3A in Pan-Cancer

**FIGURE 3:**
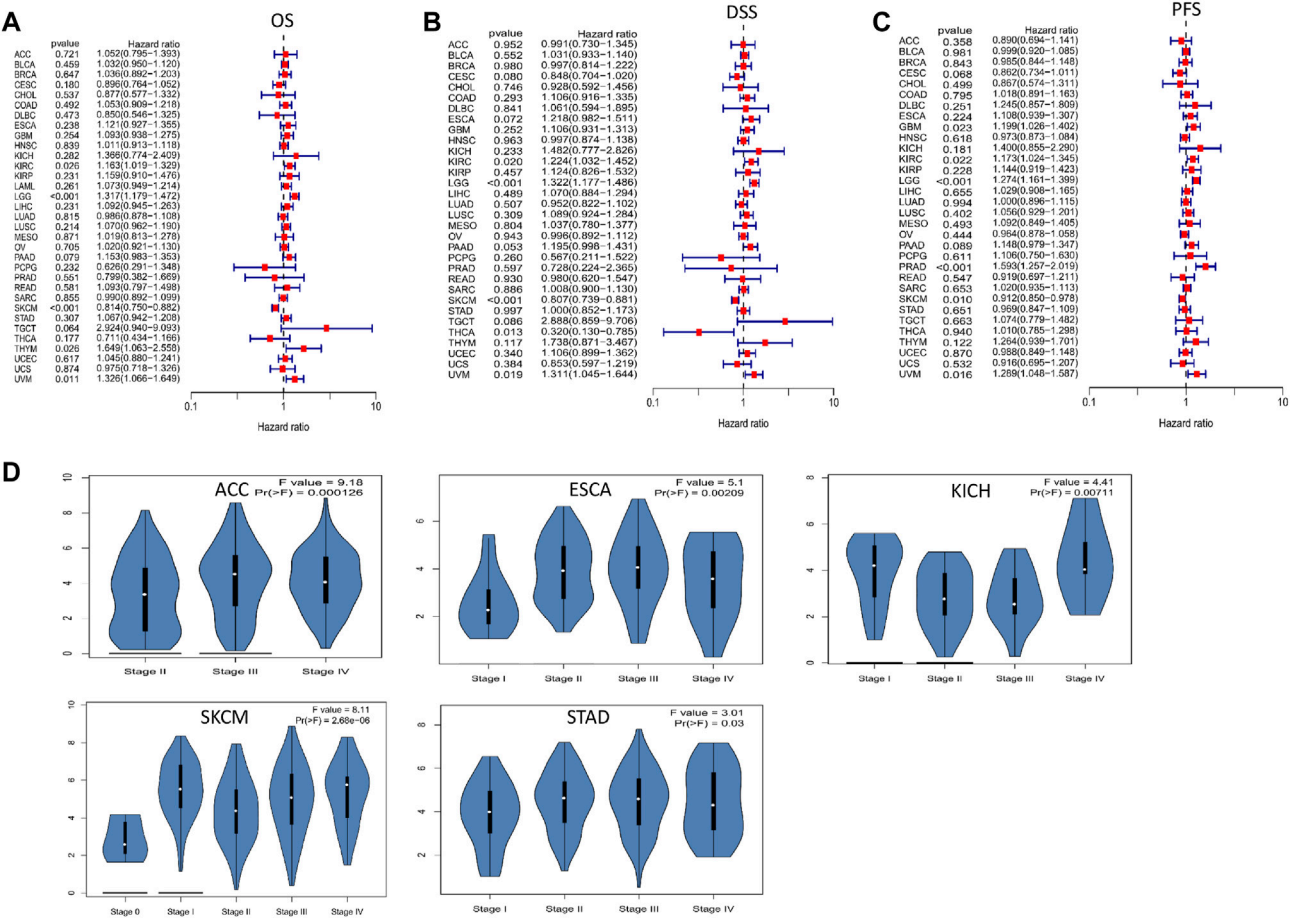
The association between FCGR3A expression levels and prognosis and tumor pathological stage. **(A)** Overall survival (OS); **(B)** Disease specific survival (DSS); **(C)** Progression-free survival (PFS); **(D)** Differential expression of FCGR3A in different stages.

To evaluate the effect of FCGR3A expression on prognosis, we conducted univariate Cox regression analysis to analyze the relationship between FCGR3A expression and OS, DSS and PFS in TCGA pan-cancer. The results are presented in Figures 3A–C, in terms of overall survival (OS), FCGR3A was an independent risk prognostic factor in KIRC (*p* = 0.026, HR = 1.163), LGG (*p* < 0.001, HR = 1.317), thymoma (THYM) (*p* = 0.026, HR = 1.649) and uveal melanoma (UVM) (*p* = 0.011, HR = 1.326), but was a protective prognostic factor in SKCM (*p* < 0.001, HR = 0.814). Then, the analysis of disease-specific survival (DSS) revealed that FCGR3A had a detrimental role in KIRC (*p* = 0.020, HR = 1.224), LGG (*p* < 0.001, HR = 1.322) and UVM (*p* = 0.019, HR = 1.311). Meanwhile, FCGR3A played a protective role in SKCM (*p* < 0.001, HR = 0.807) and THCA (*p* = 0.013, HR = 0.320). In the analysis of progression-free survival (PFS), FCGR3A was an independent risk prognostic factor in GBM (*p* = 0.023, HR = 1.199), KIRC (*p* = 0.022, HR = 1.173), LGG (*p* < 0.001, HR = 1.274), PRAD (*p* < 0.001, HR = 1. 593) and UVM (*p* = 0.016, HR = 1.289), while was a protective prognostic factor in SKCM (*p* = 0.010, HR = 0.912). It was suggested that FCGR3A is mainly a poor prognostic indicator in KIRC, LGG and UVM, on the contrary, it is also a favorable prognostic factor in SKCM.

We also examined the differential expression of FCGR3A in patients with different tumor types based on the main pathological stage and found that FCGR3A expression was only related to tumor stage in 5 cancers, including ACC, ESCA, KICH, SKCM, and STAD (Figure 3D). Notably, differences in FCGR3A expression mainly occurred between stages I-II or II-III. However, we did not obtain significant differences in other tumor types.

### Correlation Analysis of FCGR3A Expression and Tumor Immune Microenvironment

**FIGURE 4:**
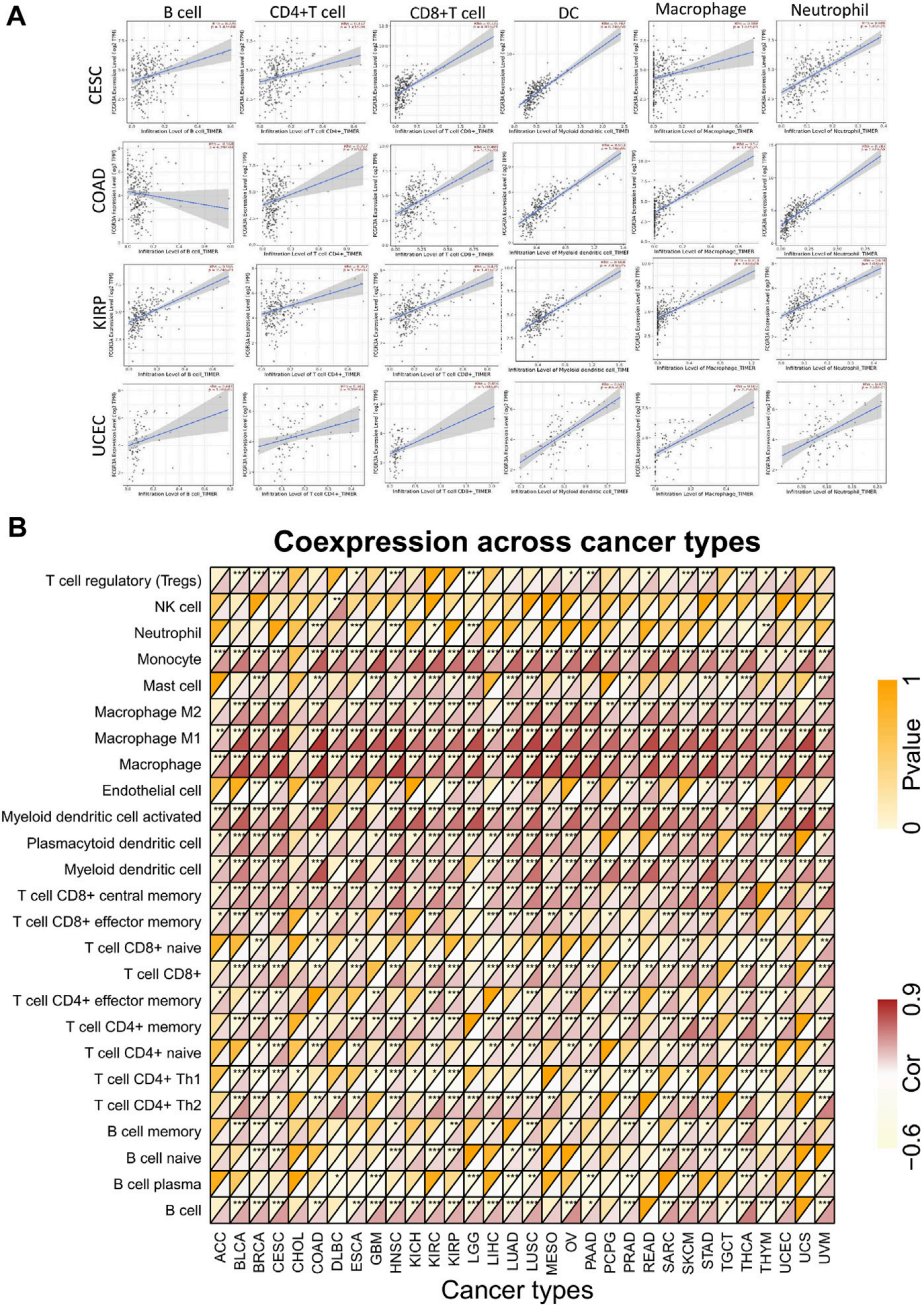
Correlation analysis of FCGR3A expression with immune cells infiltration. **(A)** The scatter plot showed a correlation between FCGR3A and the levels of infiltration of six major immune cells in CESC, COAD, KIRP, and UCEC; **(B)** Heat map showed the relationship between FCGR3A expression and 25 immune cell markers. CESC, cervical squamous cell carcinoma and endocervical adenocarcinoma; COAD, colon adenocarcinoma; KIRP, kidney renal papillary cell carcinoma; UCEC, uterine corpus endometrial carcinoma. *p < 0.05; **p < 0.01; ***p < 0.001.

Tumor infiltrating immune cells are essential components of the tumor microenvironment and play a crucial role in the modulation of tumor initiate development and immune checkpoint response (Fridman et al., 2011). Here, we first analyzed the relationship between FCGR3A expression and the infiltration levels of six common immune cells (B cells, CD4+T cells, CD8+T cells, DC cells, macrophages, and neutrophils), and observed a positive correlation in the vast majority of tumor types. The scatter diagram of correlation analysis between FCGR3A and immune infiltrating cells of each tumor can be obtained directly through the TIMER algorithm. The four highest correlation coefficients, including CESC, COAD, KIRP, and UCEC, are illustrated in Figure 4A. Moreover, we also analyzed the correlation between FCGR3A expression and 25 immune cell markers to identify potential subtypes of infiltrating immune cells. As shown in Figure 4B, FCGR3A expression level was significantly positively correlated with most of the immune cell markers, among which macrophages, monocytes, and myeloid dendritic cells were the three immune cell types most closely related to FCGR3A expression.

**FIGURE 5:**
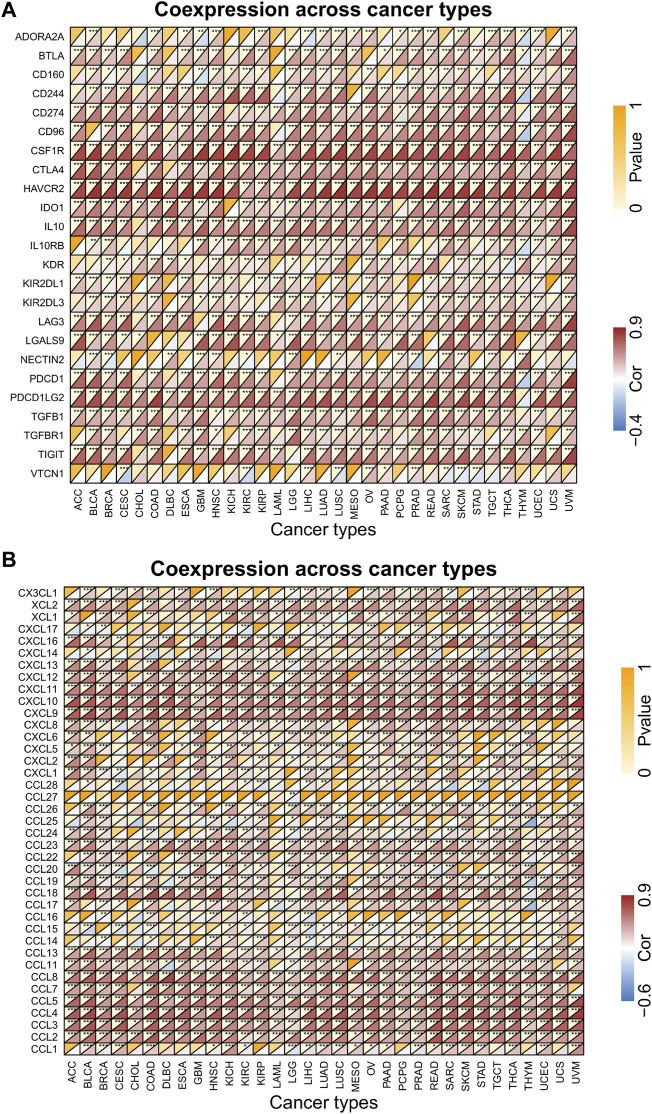
Correlation analysis of FCGR3A expression with immune-related genes. **(A)** immunosuppressive related genes; **(B)** chemokine genes. *p < 0.05; **p < 0.01; ***p < 0.001.

Gene co-expression analysis was performed to explore the relationship between FCGR3A and the expression levels of immunosuppressive genes and chemokines in 33 TCGA-cancers. As shown in Figure 5, FCGR3A expression showed significant positive correlation with almost all immunosuppressive genes (Figure 5A) and chemokine genes (Figure 5B) in pan-cancer. Therefore, these results demonstrated that FCGR3A expression affects tumor immunity in different ways.

### Analysis of Genetic Alteration Status of FCGR3A and the Relationship Between FCGR3A and DNA Mismatch Repair Genes

**FIGURE 6:**
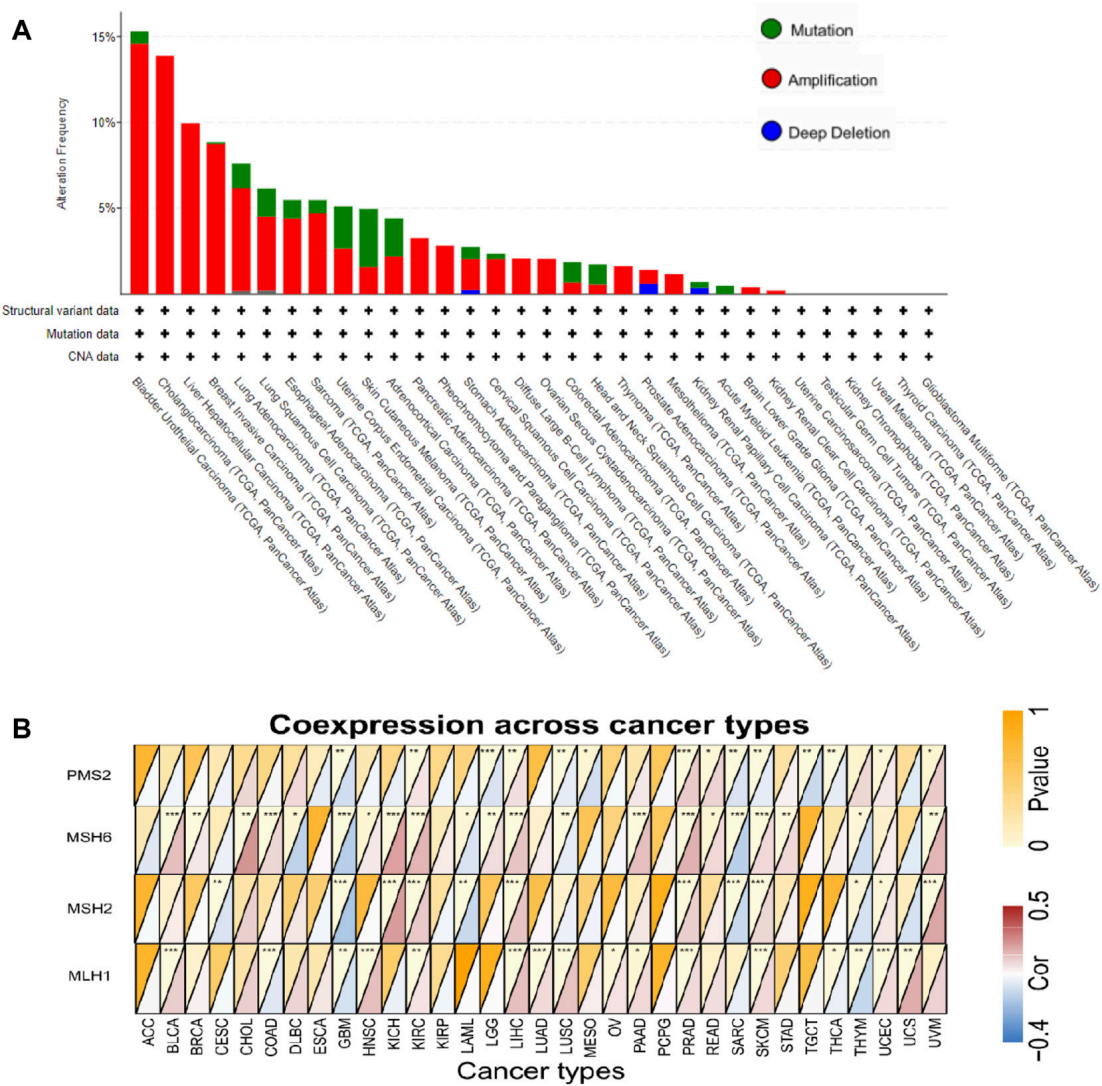
Mutation characteristics of FCGR3A in different TCGA tumors and its relationship with DNA mismatch repair genes (MMRs). **(A)** Alteration frequency of FCGR3A mutation types in different tumors; **(B)** Heat maps showed the association of APOC1 expression with expression levels of four MMRs genes in various cancers. *p < 0.05; **p < 0.01; ***p < 0.001.

The genetic alteration status of FCGR3A in various tumor samples in the TCGA cohort is shown in Figure 6A. It was not difficult for us to find that “amplification” was the main mutation type in most tumors. The alteration frequency of FCGR3A was the highest in bladder urothelial carcinoma (>15%), with “amplification” accounting for >90%. All cholangiocarcinoma, liver hepatocellular carcinoma, pancreatic adenocarcinoma pheochromocytoma and paraganglioma, diffuse large B-cell lymphoma, ovarian serous cystadenocarcinoma, thymoma, and mesothelioma cases with genetic alteration had FCGR3A amplification. The “mutation” type of FCGR3A was the dominant type in skin cutaneous melanoma, with a frequency of approximately 3%. It was noteworthy that there was a certain proportion of FCGR3A copy number deletion in stomach adenocarcinoma, prostate adenocarcinoma, and kidney renal papillary cell carcinoma.

Mismatch repair (MMR) is a post-replicative repair mechanism, which is critical for maintaining genomic fidelity (Loeb, 2001). As shown in Figure 6B, FCGR3A was positively correlated with the expression of four MMR genes (MLH1, MSH2, MSH6, and PMS2) in most tumor types, while FCGR3A showed negative correlation with MMR gene expression in CESC, GBM, LAML, sarcoma (SARC), mesothelioma (MESO), THCA and THYM. In addition, no statistical difference was observed in ACC, ESCA, KIRP, and pheochromocytoma and paraganglioma (PCPG) (*p* > 0.05).

### Association of FCGR3A Expression With DNA Methylation

**FIGURE 7:**
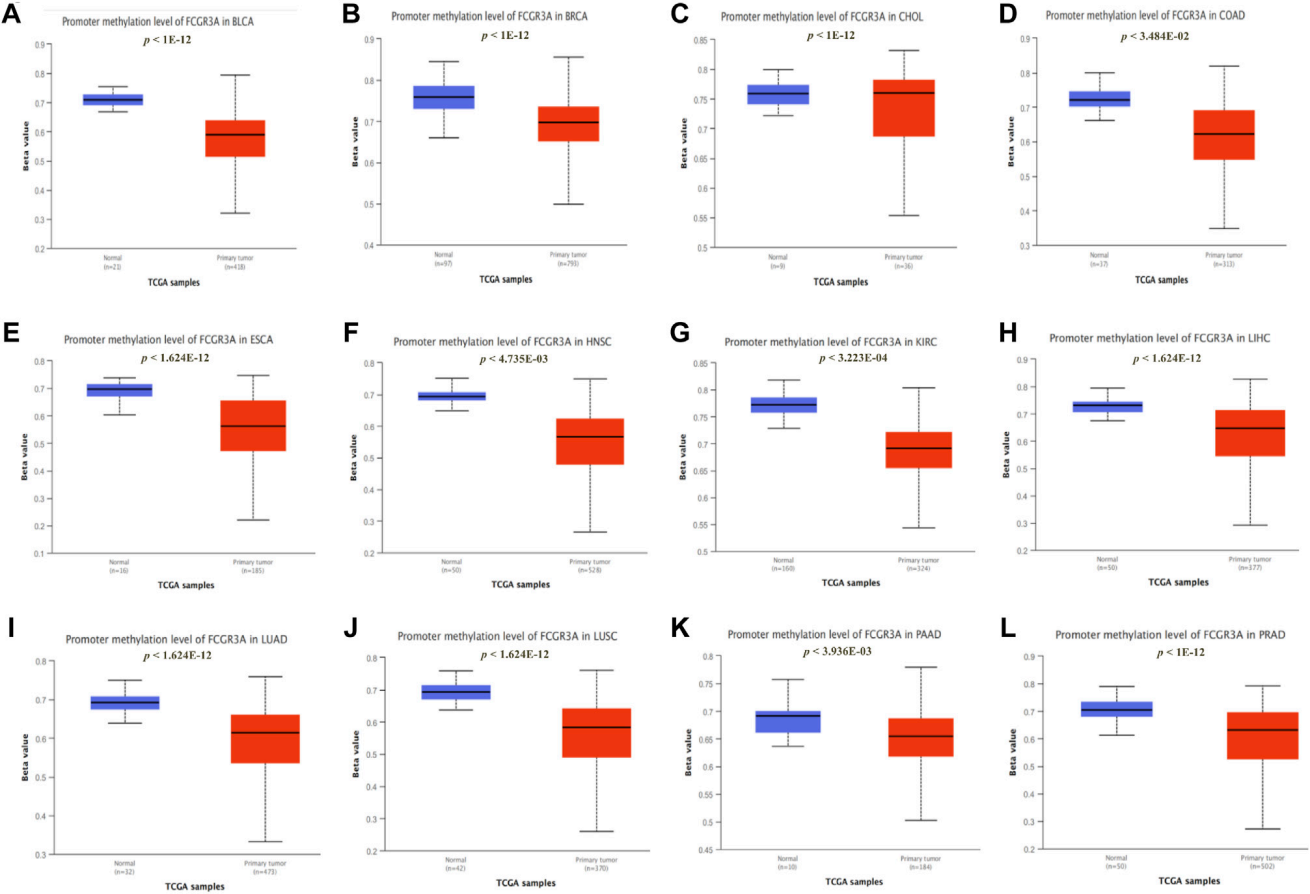
Correlation between FCGR3A expression and gene promoter methylation. **(A)** Bladder urothelial carcinoma (BLCA); **(B)** Breast invasive carcinoma (BRCA); **(C)** Cholangiocarcinoma (CHOL); **(D)** Colon adenocarcinoma (COAD); **(E)** Esophageal carcinoma (ESCA); **(F)** Head and neck squamous cell carcinoma (HNSC); **(G)** Kidney renal clear cell carcinoma (KIRC); **(H)** liver hepatocellular carcinoma (LIHC); **(I)** Lung adenocarcinoma (LUAD); **(J)** Lung squamous cell carcinoma (LUSC); **(K)** Pancreatic adenocarcinoma (PAAD); **(L)** Prostate adenocarcinoma (PRAD).

A large number of studies have demonstrated that promoter methylation leads to the inactivation of tumor suppressor genes, which is an important mechanism of tumor occurrence and development (Qureshi et al., 2010). Therefore, we used the UALCAN dataset to analyze the methylation levels of the FCGR3A promoter in different tumors and normal tissues, which determined that the methylation levels of the FCGR3A promoter in 12 tumors including BLCA, BRCA, CHOL, COAD, ESCA, HNSC, KIRC, LIHC, lung adenocarcinoma (LUAD), LUSC, PAAD, and PRAD were significantly lower than those in normal tissues (Figures 7A–L).

### PPI Network and KEGG/GO Enrichment Analysis of FCGR3A Related Genes

**FIGURE 8:**
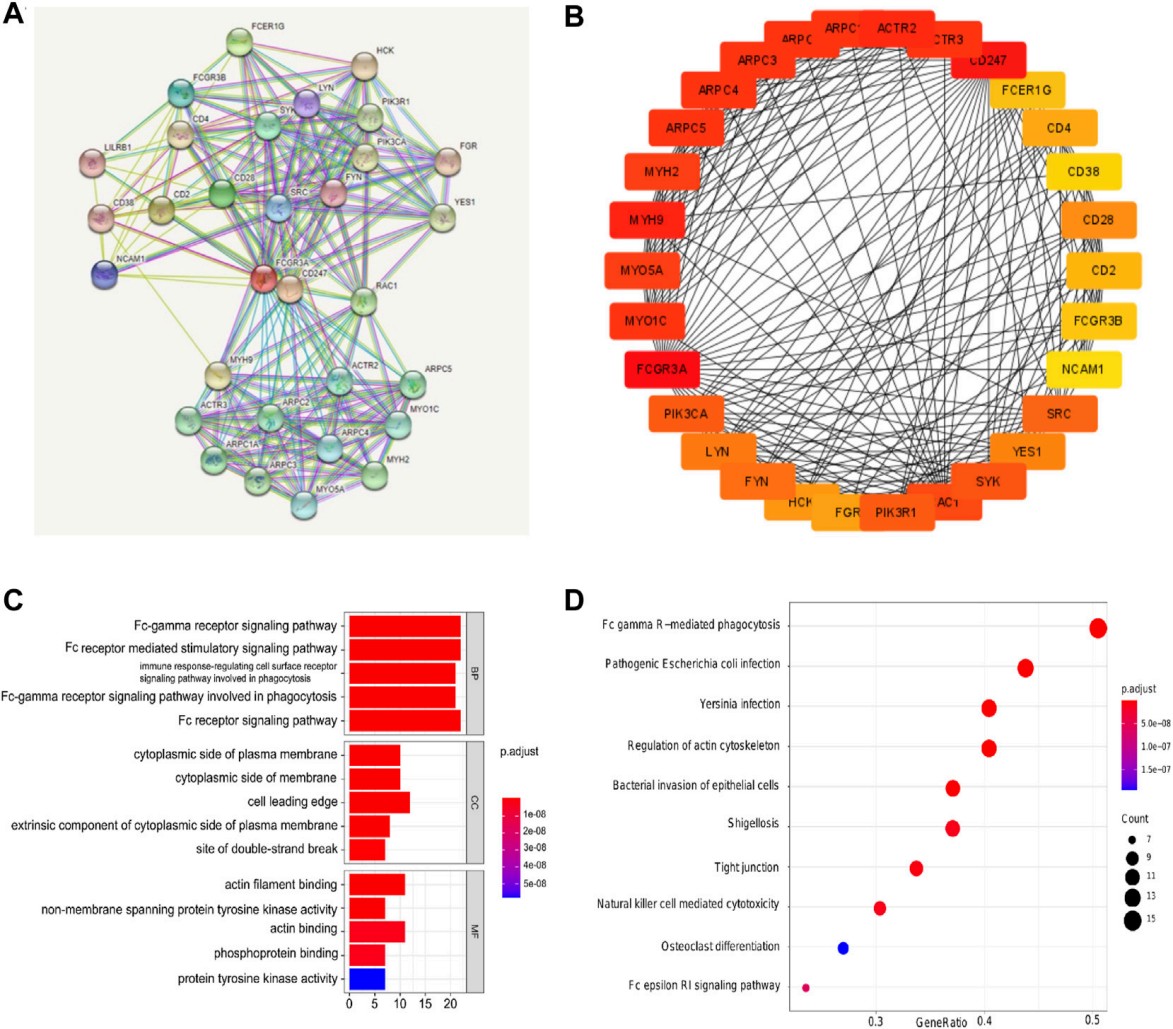
Protein-protein interaction (PPI) network, gene ontology (GO) analysis, and Kyoto Encyclopedia of Genes and Genomes (KEGG) analysis of FCGR3A-related genes. **(A,B)** PPI network; **(C)** GO analyses; **(D)** KEGG analysis.

In order to further explore the molecular mechanism of FCGR3A gene in tumorigenesis, we obtained a total of 30 FCGR3A targeted binding proteins by using the STRING tool. Figure 8A showes the interaction network of these proteins. Cytoscape software was utilized to further visualized the PPI network (Figure 8B). The results of GO and KEGG pathway analysis are presented in Figures 8C,D. The GO enrichment analysis consisted of three parts: biological process (BP), cellular component (CC), and molecular function (MF). The BP primarily included the Fc receptor signaling pathway, Fc receptor mediated stimulatory signaling pathway, Fc-gamma receptor signaling pathway, Fc-gamma receptor signaling pathway involved in phagocytosis, and immune response-regulating cell surface receptor signaling pathway involved in phagocytosis. The CC was mainly covered with cell leading edge, actin filament, site of DNA damage, site of double-strand break and Arp2/3 protein complex. The MF was primarily enriched in actin binding, actin filament binding, protein tyrosine kinase activity, phosphoprotein binding, and non-membrane spanning protein tyrosine kinase activity. The KEGG pathway was associated with Fc gamma mediated phagocytosis, pathogenic *Escherichia coli* infection, regulation of actin cytoskeleton, *Yersinia* infection, and bacterial invasion of epithelial cells.

### Correlation Between FCGR3A and Cancer Pathway

**FIGURE 9:**
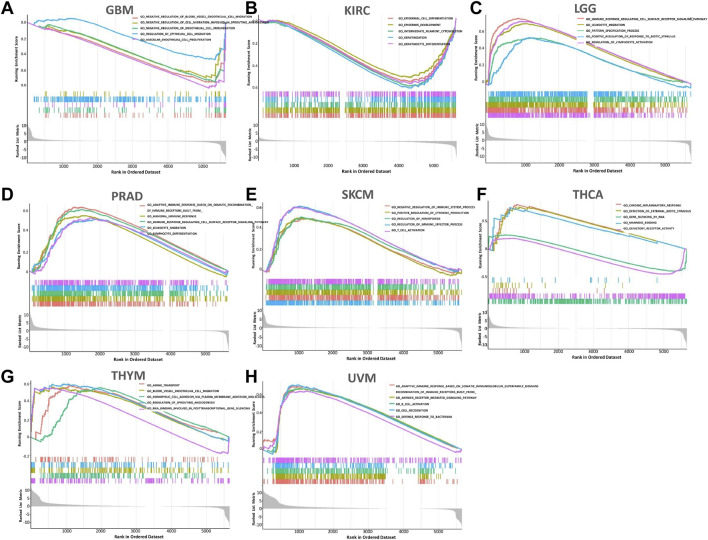
The 5 most relevant signaling pathways of FCGR3A’s GSEA in the GO dataset. **(A)** Glioblastoma multiforme (GBM); **(B)** Kidney renal clear cell carcinoma (KIRC); **(C)** brain lower grade glioma (LGG); **(D)** Prostate adenocarcinoma (PRAD); **(E)** Skin cutaneous melanoma (SKCM); **(F)** Thyroid carcinoma (THCA); **(G)** Thymoma (THYM); **(H)** Uveal melanoma (UVM).

In order to observe the expression and enrichment status of FCGR3A in GO pathway sets, we divided the human tumor samples into high expression and low expression group according to the median FCGR3A expression and analyzed the enrichment of the GO signaling pathway in the two groups by GSEA. The top 5 signaling pathways with the most significant enrichment in the eight tumors (including GBM, KIRC, LGG, PRAD, SKCM, THCA, THYM, and UVM) are listed in Figures 9A–H. It was found that negative regulation of blood vessel endothelial cell migration, epidermal cell differentiation, immune response regulating cell surface receptor signaling pathway, adaptive immune response based on somatic recombination, immune receptors built from, humoral immune response, immune response regulating cell surface receptor signal pathway, negative regulation of immune system process, positive regulation of cytokine production, chronic inflammatory response, erection of external biotic stimulus and adaptive immune response based on somatic were described as the most abundant GO pathways in eight tumors whose prognosis was associated with APOC1 expression. This suggested that FCGR3A is extensively implicated in the negative regulation of tumor angiogenesis and the regulation of cancer immune signaling pathways.

### Relationship Between FCGR3A Expression and Drug Sensitivity

**FIGURE 10:**
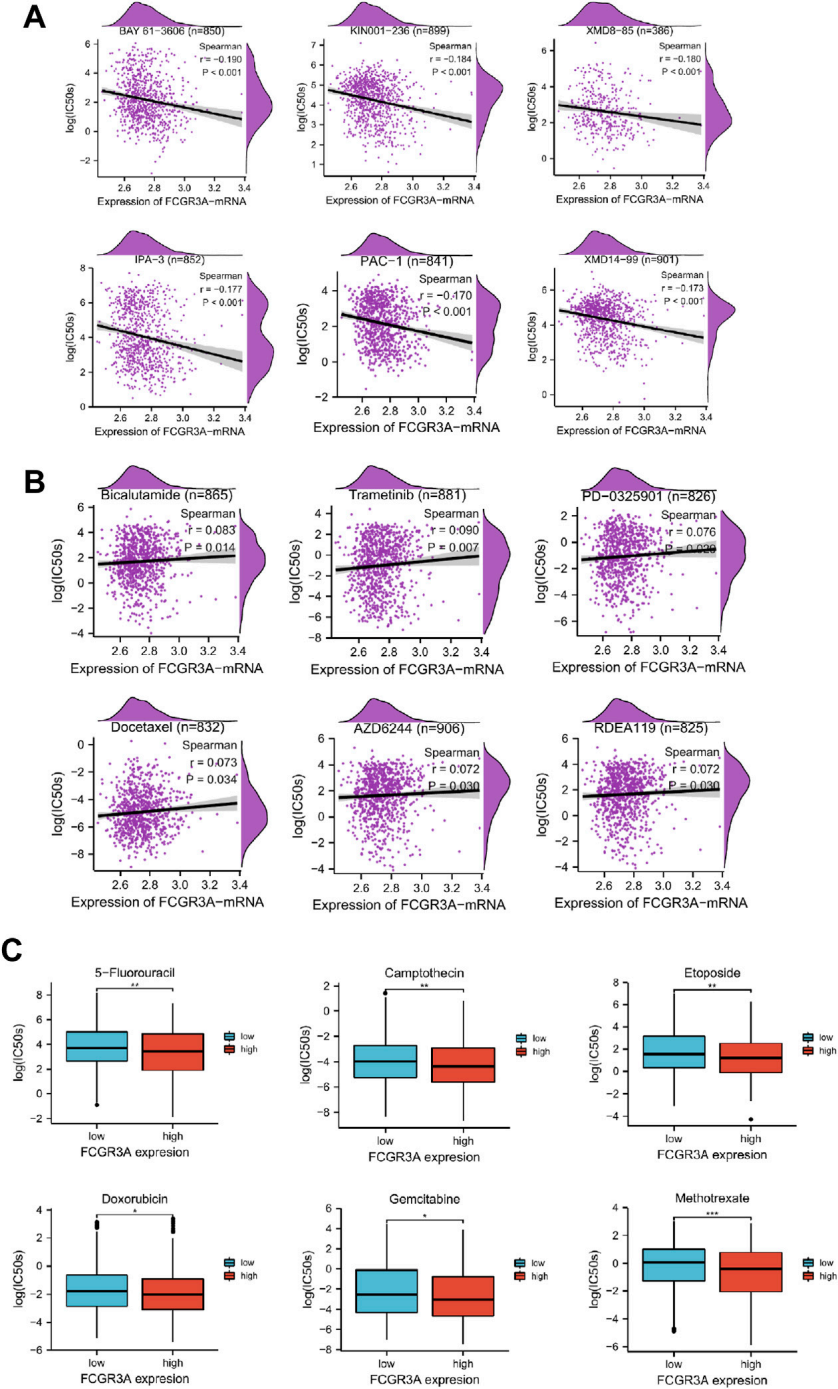
Relationship between FCGR3A expression and drug sensitivity. **(A)** The top six negatively correlated. **(B)** The only six positively correlated. **(C)** The difference of drug sensitivity of six commonly used anticancer drugs (5-fluorouracil, Camptothecin, Etoposide, Doxorubicin, Gemcitabine, and Methotrexate) in high and low FCGR3A expression groups was shown in the forms of boxplot charts. *p < 0.05; **p < 0.01; ***p < 0.001.

Genetic alterations affect the drug sensitivity of cancer to clinical treatment and therefore are potential biomarkers for drug screening. Therefore, we question the association between mRNA expression levels of FCGR3A and patient sensitivity to antitumor drugs. Based on the GDSC database, we performed a correlation analysis between gene expression level and drug sensitivity of 265 drugs across 963 cell lines, and a total of 158 drugs were identified to be associated with FCGR3A expression. Figures 10A,B exhibites the six drugs with the strongest negative correlation and the six drugs with the strongest positive correlation respectively. The IC50s of BYA 61-3606 (r = −0.190, *p* < 0.001), KIN001-236 (r = −0.184, *p* < 0.001), XDM8-85 (r = −0.180, *p* < 0.001), IPA-3 (r = −0.177, *p* < 0.001), PAC-1 (r = −0.170, *p* < 0.001) and XMD14-99 (r = −0.173, *p* < 0.001) were negatively correlated with FCGR3A expression (Figure 10A). Additionally, the IC50s of six drugs (only 6), including Bicalutamide (r = 0.083, *p* = 0.014), Trametinib (r = 0.090, *p* = 0.007), PD-0325901 (r = 0.076, *p* < 0.020), Doxetaxel (r = 0.073, *p* = 0.034), AZD6244 (r = 0.072 *p* = 0.030) and READ119 (r = 0.072, *p* = 0.030), were positively correlated with FCGR3A expression (Figure 10B). Complete drug sensitivity analysis results are shown in Supplementary Table S1. As shown in Figure 10C, six commonly used anticancer drugs, such as 5-fluorouracil, Camptothecin, Etoposide, Doxorubicin, Gemcitabine, and Methotrexate have lower IC50 values (better efficacy) in patients with high FCGR3A expression. These results confirmed our hypothesis that the expression level of FCGR3A interacts with the sensitivity of antitumor drugs.

## Discussion

FCGR3A is the encoding gene of CD16a, and the up-regulation of FCGR3A results in high expression of CD16a. Almost all NK cells express the low-affinity Fc γ receptor (FCγR) IIIA/CD16a. The activated receptor CD16a on the NK cell surface promotes antibot-dependent cell-mediated cytotoxicity (ADCC), which is a key effect of NK cells and tumor antigen-targeting mechanism (Nimmerjahn and Ravetch, 2008).

Many but not all studies have reported significant associations between functional polymorphisms of Fcγ RIIIA-activated receptors and antitumor mAb immunotherapy outcomes (Cartron et al., 2002; Weng and Levy, 2003; Treon et al., 2005; Calemma et al., 2012; Seidel et al., 2013; Gavin et al., 2017; Magnes et al., 2018). Unfortunately, whether FCGR3A works in different tumors through some common molecular mechanism remains to be answered. A literature search revealed limited and weak data available for a pan-cancer analysis of FCGR3A from a whole-tumor perspective. Therefore, we comprehensively examined FCGR3A in pan-cancer based on data from TCGA and GTEx databases.

We found that FCGR3A was overexpressed in 23 cancers, and IHC analysis confirmed this trend at the protein level. COX regression analysis showed that the prognosis analysis data of the FCGR3A gene proposed different conclusions for different tumors, but these correlations were only seen in a small number of tumors. High FCGR3A expression was mainly associated with poor prognoses of six cancer types (KIRC, LGG, THYM, PRAD, and UVM), while it was associated with good prognoses of SKCM and THCA. This difference may be due to discrepancies in tumor samples, so larger sample sizes may be needed to verify the above conclusions. Combined with the above results, FCGR3A is regarded as a potential biomarker or therapeutic target.

With the clinical success of cancer immunotherapy, there is an increasing need for a comprehensive understanding of tumor-immune interactions. Understanding the tumor microenvironment, including immune cell infiltration, may help decipher the mechanisms behind tumor development. The Fc receptor (FcR) plays a major role in normal immunity and anti-infection and provides the humoral immune system with cellular effectors. Previous studies have suggested that the G-protein-coupled receptors (GPCR) of chemokines can detect chemical attractants produced by bacteria in inflammatory responses and activate signaling pathways that regulate actin polymerization and allow cells to migrate to bacteria. Various receptors, such as Fcγ and C3a receptors, then bind to targets and activate signaling pathways, resulting in actin aggregation around the bacteria, forming phagocytic rings (Pan et al., 2016). An effective mechanism of tumor cell phagocytosis is mediated by the uptake of Fc receptors (FcR) by antibody-coated tumor cells. Depending on the nature of the FcR and Fc parts of the antibody, binding of the cell-associated antigen-antibody complex to FcR can trigger complement, ADCC, or phagocytosis and induce DC maturation, thereby stimulating T cell responses against the captured antigens and antitumor immunity (Rafiq et al., 2002; Schuurhuis et al., 2006; Dilillo and Ravetch, 2015).

CD8+T cells are known to regulate specific expression of tumor antigens, so they are considered as key mediators of tumor destruction and play an indispensable role in immunotherapy (Kishton et al., 2017). Clinical studies on tumor-infiltrating immune cells have identified the role of cytotoxic T cells (CTL) and tumor-associated macrophages (TAM) in some diseases (Bingle et al., 2002; Fridman et al., 2012). In addition to focusing on CD8+T cells, this study also observed a significant association between tumor FCGR3A expression and other immune cells such as CD4^+^ T cells, B cells, DC cells, macrophages, and neutrophil infiltration, although the current study was unable to establish a causal relationship. Interestingly, the relationship between FCGR3A expression and certain immune cell markers (such as Th1, Th2, M1/M2 macrophages, mast cells, monocytes, Tregs, and so on) does not always follow the general trend (Figure 6C), suggesting specific interactions between FCGR3A and certain immune cell subtypes. In addition, our study also revealed the co-expression of FCGR3A and immunosuppression-related genes and chemokine genes. These results indicate that the expression level of FCGR3A can affect the tumor immune microenvironment, which will provide a new reference for the prognosis of immune checkpoint inhibitors (ICIs) treatment.

Mismatch repair (MMR) genes are efficient guardians of genomic integrity and stability, and the presence of MMR gene mutations can predict tumor patients’ sensitivity to immune checkpoint blocking therapy (Le et al., 2017). According to our results, the expression of FCGR3A was positively correlated with the expression of MMRs genes in most tumors, suggesting that FCGR3A may maintain the viability of tumor cells by up-regulating DNA mis-match repair-related genes.

The mutation analysis found that the alteration frequency of FCGR3A in bladder urothelial carcinoma was the highest, and the main type is the amplification mutation. This enriched our understanding of the functionality of FCGR3A. Furthermore, disruption of DNA methylation patterns is a relatively common feature in cancer and is associated with various developmental defects and tumorigenesis (Fernandez et al., 2012). Feinberg, A.P et al. also reported significant hypommethylation in cancer genes compared with normal corresponding cells (Feinberg and Vogelstein, 1983). Similarly, our study reached the same conclusion, suggesting that FCGR3A may influence DNA methylation and promote tumor development, although the detailed mechanism is still unknown.

Through GO and KEGG pathway analysis, we further clarified FCGR3A’s involvement in a variety of biological processes, molecular functions, and cellular components, mainly including the Fc receptor signaling pathway, immune response-regulating cell surface receptor signaling pathway involved in phagocytosis, humoral immunity, and pathogen infection. Using GSEA enrichment analysis, it was found that FCGR3A was mainly enriched in pathways related to negative regulation of angiogenesis, epidermal cell differentiation, chronic inflammation, and adaptive immune response.

The analysis of basic gene expression can reveal the relationship between cell’s resting physiological state and drug sensitivity, which is valuable for the analysis of drug action mechanism. Through drug sensitivity analysis, we found that high FCGR3A expression was negatively correlated with IC50 values of most anticancer drugs (i.e., drug response was well). This is consistent with previous findings (Vuong et al., 2014; Salvadores et al., 2020) that the level of gene expression is a predictor of drug response. It is indicated that the measurement of FCGR3A expression level can be used as a reliable indicator of clinical treatment, highlighting the potential of FCGR3A as an anticancer drug target. Even if it is not a direct target of a compound, it may play a crucial role in the processes before and after the compound binds to its target, and variations in its expression may underlie individual differences in drug responses (Vuong et al., 2014). These results will help us better understand how drug-target interactions can benefit cancer treatment, and have important implications for guiding the combination therapy in patients with advanced or recurrent tumor, and guiding the drug selection in patients with multiline treatment resistance.

In searchable articles reports, most of studies on FCGR3A (CD16a) have focused on the pathogenesis, diagnosis, and treatment of inflammatory diseases such as IgG immune complexes (including rheumatoid arthritis and systemic lupus erythematosus), and little attention has been paid to cancer. In 2020, Hofmann L et al. confirmed that CD16-positive exosomes could be used as an indicator of immunosuppressive grade of HNSC: the later the tumor stage or the more aggressive the tumor, the higher the CD16 level of total exosomes in patients (Hofmann et al., 2020). Sconocchia G et al. found that tumor invasion of FcγRIII (CD16) + bone marrow cells is associated with improved survival in colorectal cancer patients (Sconocchia et al., 2011). And Zhang W et al. indicated that relatively increased CD16 + monocytes contribute to the pro-tumor microenvironment of T cell non-Hodgkin lymphoma (Zhang et al., 2020). This provides an idea for our study. The significance of our work is to prospectively reveal the interaction of FCGR3A in the tumor microenvironment and provide insights based on bioinformatics and computational biology for further understanding the role of FCGR3A in tumor metabolism and immune regulation.

However, there are still had several limitations in our study. First, the results of this study were acquired solely from bioinformatics analysis, lacking actual experimental or clinical data. Second, tumor tissue information is mainly derived from the collection of large amounts of microarray and sequencing data from public databases, and cell-level analysis of immune cell markers may introduce systematic bias. Third, although we identified that FCGR3A expression was associated with survival in some tumor patients and affected immune cell infiltration, we were unable to prove a clear causal relationship between FCGR3A altering immune infiltration and affecting patient survival. The role of FCGR3A in cancer needs to be further explored and verified through biological experiments in the future.

In conclusion, FCGR3A not only affects the prognosis of cancer patients and regulates the immune microenvironment, but also is a strong predictor for anticancer drug response, suggesting that FCGR3A may be an immunocarcinogenic molecule and can be used as a promising biomarker to provide a direction for immune-based anti-tumor strategies.

## Data Availability

The original contributions presented in the study are included in the article/Supplementary Material, further inquiries can be directed to the corresponding author.

## Supplementary Material

Supplementary Figure S1 The expression of FCGR3A in different TCGA cancer types was analyzed using TIMER **(A)** and GEPIA2 **(B)** respectively.

Supplementary Figure S2 The expression of FCGR3A in pan-cancer single cell lines was analyzed using GDSC database **(A)** and the cancerSCEM website **(B)** respectively.

Supplementary Table S1 Spearman correlation analysis between FCGR3A expression and all the 265 drugs IC50 in the GDSC database.

## Glossary

ACC: adrenocortical carcinoma
ADCC: antibot-dependent cell-mediated cytotoxicity
ADCP: antibody-dependent cellular phagocytosis
BP: biological process
BLCA: bladder urothelial carcinoma
BRCA: breast invasive carcinoma
CAN: copy number alteration
CC: cellular component
CESC: cervical squamous cell carcinoma and endocervical adenocarcinoma
CHOL: cholangiocarcinoma
COAD: colon adenocarcinoma
CLL: chronic lymphocytic leukemia
CRC: coloretal cancer
CPTAC: Clinical Proteomic Tumor Analysis Consortium
CI: confidence interval
CTL: cytotoxic T cells
DC: dendritic cell
DLBC: lymphoid neoplasm diffuse large B-cell lymphoma
DSS: disease-specific survival
ESCA: esophageal carcinoma
FCGR3A: Fc gamma receptors 3A
GBM: glioblastoma multiforme
GDSC: Genomics of Drug Sensitivity in Cancer
GEPIA: Gene Expression Profiling Interactive Analysis
GTEx: Genotype-Tissue Expression
GO: Gene Ontology
GPCR: G-protein-coupled receptors
HER2: human epidermalgrowth factor receptor-2
HNSC: head and neck squamous cell carcinoma
HPA: Human Protein Atlas
HR: hazard ratio
ICI: Immune checkpoint inhibitor
IC50: half maximal inhibitory concentration
IHC: immunohistonchemistry
KEGG: Kyoto Encyclopedia of Genes and Genomes
KICH: kidney chromophobe
KIRC: kidney renal clear cell carcinoma
KIRP: kidney renal papillary cell carcinoma
LAML: acute myeloid leukemia
LGG: brain lower grade glioma
LIHC: liver hepatocellular carcinoma
LUAD: lung adenocarcinoma
LUSC: lung squamous cell carcinoma
MESO: mesothelioma
MMR: DNA mis-match repair genes
MF: molecular function
OV: ovarian serous cystadenocarcinoma
OS: overall survival
PAAD: pancreatic adenocarcinoma
PCPG: pheochromocytoma and paraganglioma
PD-L1: programmed cell death-Ligand 1
PFI: progression-free interval
PFS: progression-free survival
PRAD: prostate adenocarcinoma
PPI: protein–protein interaction
READ: rectum adenocarcinoma
SARC: sarcoma
SKCM: skin cutaneous melanoma
STAD: stomach adenocarcinoma
TAM: tumor-associated macrophages
TCGA: The Cancer Genome Atlas
TGCT: testicular germ cell tumors
THCA: thyroid carcinoma
THYM: thymoma
TIMER: Tumor Immune Estimate Resources
UCEC: uterine corpus endometrial carcinoma
UCS: uterine carcinosarcoma
UVM: uveal melanoma
