# Supplementary figures and images for: Integrative Pan-Cancer Analysis Confirmed that FCGR3A is a Candidate Biomarker Associated With Tumor Immunity
Source: Front Pharmacol. 2022 May 20;13:900699. doi: 10.3389/fphar.2022.900699 (PMC9163829; doi:10.3389/fphar.2022.900699)

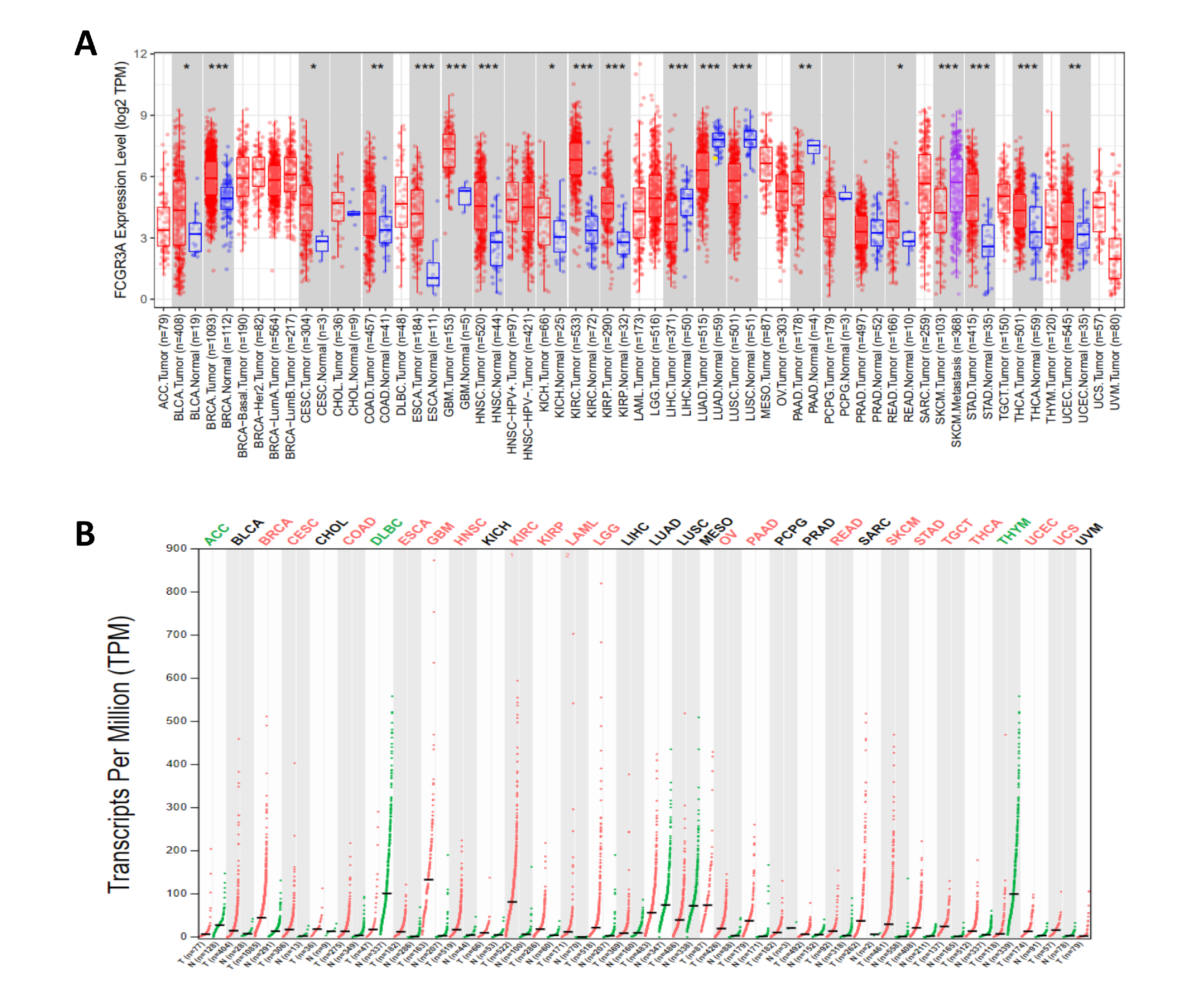

Supplement: Supplementary file 1 [file Image1.TIFF]

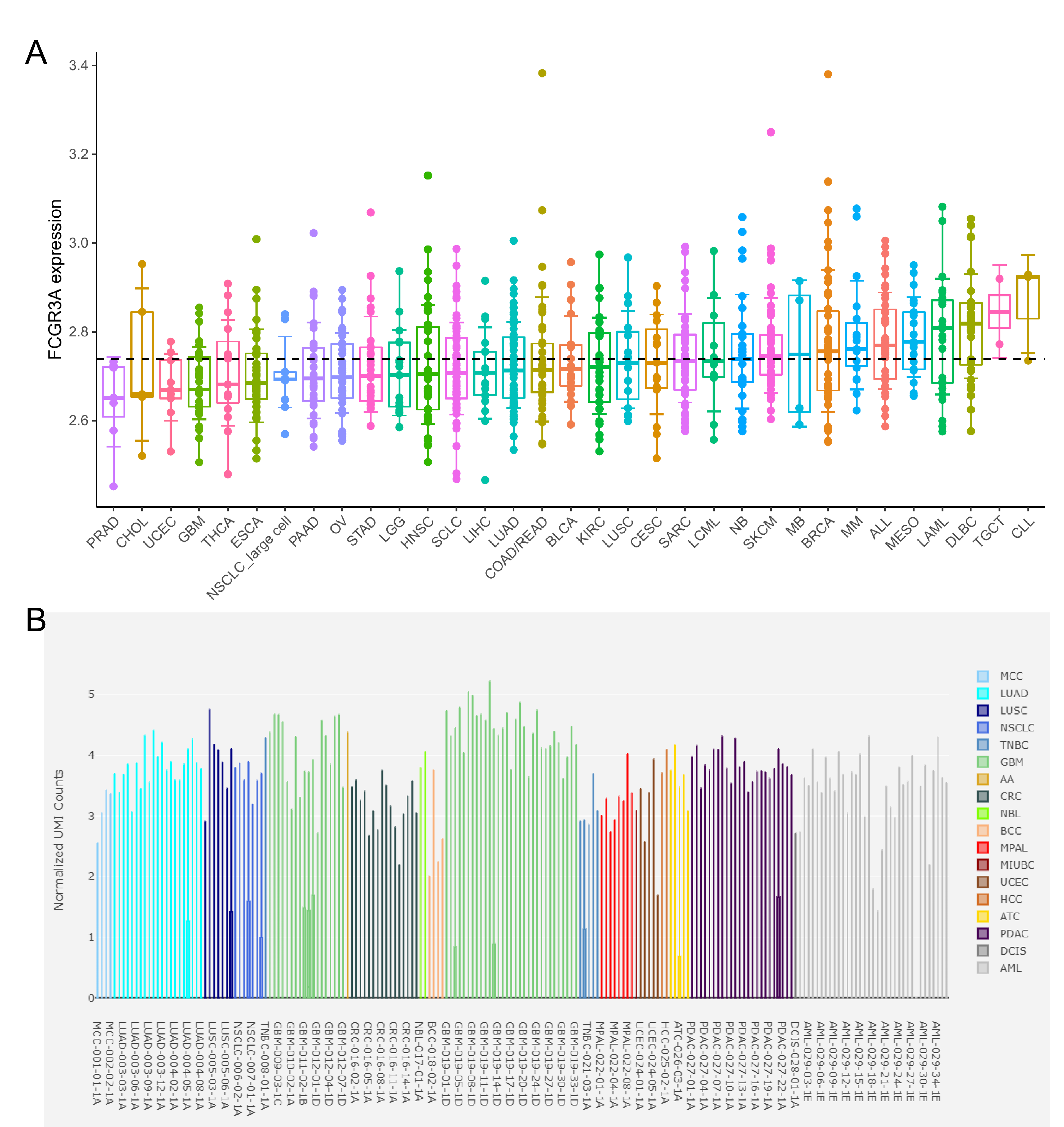

Supplement: Supplementary file 2 [file Image2.TIFF]
